# The Clinical Presentation of Puumala Hantavirus Induced Hemorrhagic Fever with Renal Syndrome Is Related to Plasma Glucose Concentration

**DOI:** 10.3390/v13061177

**Published:** 2021-06-20

**Authors:** Johanna Tietäväinen, Satu Mäkelä, Heini Huhtala, Ilkka H. Pörsti, Tomas Strandin, Antti Vaheri, Jukka Mustonen

**Affiliations:** 1 Faculty of Medicine and Health Technology, Tampere University, 33520 Tampere, Finland; satu.m.makela@pshp.fi (S.M.); ilkka.porsti@tuni.fi (I.H.P.); jukka.mustonen@tuni.fi (J.M.); 2 Department of Internal Medicine, Tampere University Hospital, 33520 Tampere, Finland; 3 Faculty of Social Sciences, Tampere University, 33520 Tampere, Finland; heini.huhtala@tuni.fi; 4 Department of Virology, Medicum, University of Helsinki, 00290 Helsinki, Finland; tomas.strandin@helsinki.fi (T.S.); antti.vaheri@helsinki.fi (A.V.)

**Keywords:** puumala hantavirus, hyperglycemia, capillary leakage, thrombocytopenia, AKI, endothelial damage

## Abstract

Puumala hantavirus (PUUV) causes a hemorrhagic fever with renal syndrome characterized by thrombocytopenia, increased capillary leakage, and acute kidney injury (AKI). As glucosuria at hospital admission predicts the severity of PUUV infection, we explored how plasma glucose concentration associates with disease severity. Plasma glucose values were measured during hospital care in 185 patients with PUUV infection. They were divided into two groups according to maximum plasma glucose concentration: P-Gluc < 7.8 mmol/L (*n* = 134) and P-Gluc ≥ 7.8 mmol/L (*n* = 51). The determinants of disease severity were analyzed across groups. Patients with P-Gluc ≥7.8 mmol/L had higher hematocrit (0.46 vs. 0.43; *p* < 0.001) and lower plasma albumin concentration (24 vs. 29 g/L; *p* < 0.001) than patients with P-Gluc < 7.8 mmol/L. They presented with higher prevalence of pulmonary infiltrations and pleural effusion in chest radiograph, higher prevalence of shock and greater weight change during hospitalization. Patients with P-Gluc ≥ 7.8 mmol/L were characterized by lower platelet count (50 vs. 66 × 10^9^/L; *p* = 0.001), more severe AKI (plasma creatinine 272 vs. 151 µmol/L; *p* = 0.001), and longer hospital treatment (8 vs. 6 days; *p* < 0.001) than patients with P-Gluc < 7.8 mmol/L. Plasma glucose level is associated with the severity of capillary leakage, thrombocytopenia, inflammation, and AKI in patients with acute PUUV infection.

## 1. Introduction

Hantaviruses cause two clinical disease entities. Hemorrhagic fever with renal syndrome (HFRS) found in Eurasia is caused by Hantaan, Puumala and Dobrava viruses. Hantavirus cardiopulmonary syndrome (HCPS) present in the Americas is caused by Sin Nombre, Andes and related hantaviruses. Seoul virus that causes HFRS is found worldwide [1].

Puumala virus (PUUV) is the only human pathogenic hantavirus in Finland. It is a member of the *Orthohantavirus* genus in the *Hantaviridae* family, order *Bunyavirales* and it is carried by the bank vole (*Myodes glareolus*). PUUV-induced HFRS is characterized by thrombocytopenia, increased capillary leakage, and acute kidney injury (AKI).

The clinical picture of PUUV infection varies from asymptomatic infection to severe disease, but most cases have a mild course. In typical forms of infection, five distinct phases can be recognized: febrile, hypotensive, oliguric, polyuric and convalescent. Patients usually arrive at the hospital at the hypotensive phase when capillary leakage prevails. Patients may have pulmonary infiltrations and pleural effusion, pericardial effusion, and retroperitoneal edema [1]. Of the hospitalized patients, less than 5% have circulatory shock. As markers of renal involvement, most patients have transient proteinuria and hematuria. The hypotensive phase is followed by oliguria and AKI. About 6% need transient dialysis treatment. Polyuria, that can be substantial, is a sign of recovery [2]. Bleeding diathesis is rare and case fatality is less than 0.5% [2]. Host genetic factors influence the clinical course of the disease [3].

The impact of hyperglycemia on the outcome of hospitalized patients has been studied in various diseases. It is currently appreciated that hyperglycemia, whether critical illness induced or related to diabetes, is associated with poor outcome in severely ill patients [4,5]. In diabetic SARS-CoV2 patients, poorly controlled glycemic variability is associated with higher mortality as well as with higher incidence of AKI, septic shock, acute respiratory distress syndrome (ARDS) and disseminated intravascular coagulation (DIC) [6,7]. Interestingly, fasting blood glucose at admission in COVID-19 patients without previous diagnosis of diabetes is also an independent predictor for 28-day mortality in a multi-center retrospective study [7].

There is a growing body of evidence showing that acute hyperglycemia in non-diabetic patients is related to kidney injury. In patients with acute myocardial infarction, admission hyperglycemia is associated with AKI [8]. In an experimental animal model, 6-h glucose infusion caused structural and functional changes in kidney glomeruli and tubular epithelial cells, and induced structural changes in small vessels [9]. Of these, renal tubular injury appeared to be most prominent. In addition, transient hyperglycemia induced inflammation, oxidative stress and apoptosis [9].

Recently we reported that 12% of patients with PUUV infection have mild and transient glucosuria on admission to hospital. Glucosuria is associated with more severe thrombocytopenia and inflammation, as well as with higher capillary leakage and more severe AKI [10]. Histologically PUUV causes acute tubulointerstitial nephritis (ATIN), and therefore we hypothesized that more severe ATIN impaired glucose reuptake in the proximal tubule of the kidney resulting in glucosuria [10]. In that study, blood glucose concentration was slightly higher in the glucosuric patients, but in the majority of patients, it was below the kidney glucose threshold level [10]. This finding led us to investigate the association of blood glucose level with the severity of PUUV infection.

We analyzed the outcome of patients grouped according to the maximum plasma glucose concentration of either <7.8 mmol/L or ≥7.8 mmol/L during hospital treatment. According to our results, plasma glucose concentration is associated with all the known determinants of disease severity of acute PUUV infection. In addition, markers of increased capillary leakage and levels of thrombocytopenia and leukocytosis are associated dose dependently with maximum plasma glucose level.

## 2. Materials and Methods

### 2.1. Subjects

The study cohort included 185 adult patients treated in Tampere University Hospital, Finland, due to serologically confirmed acute PUUV infection during the years 1986–2017. Information of plasma glucose level during hospital care was collected retrospectively. Analyses of plasma glucose concentrations during hospital treatment were based on clinical relevance. One glucose value was found for 127 patients, two for 39 patients, and 3–10 values for 19 patients.

A detailed medical history of the patients was obtained, and all patients were carefully clinically examined. Blood pressure, heart rate and weight were measured at least daily. The daily urine output was followed during the hospital stay.

Out of the 185 patients, 69 (37%) had one or several previous diagnoses: hypertension (*n* = 19), hypercholesterolemia (*n* = 8), type 2 diabetes (*n* = 6), cardiac arrhythmia/conduction disturbance (*n* = 6), coronary heart disease (*n* = 4), rheumatoid arthritis (*n* = 3), coeliac disease (*n* = 3), operated malignancy (*n* = 3), prostate hyperplasia (*n* = 2), osteoporosis (*n* = 2), psoriasis (*n* = 2), and mental illness (*n* = 2). In addition, the following diagnoses were present in one patient each: hyperthyroidism, hypothyroidism, cerebral infarction, juvenile rheumatoid arthritis, Sjögren’s syndrome, spondyloarthropathy, Crohn’s disease, epilepsy, sarcoidosis, idiopathic thrombocytopenia, bronchial asthma and spherocytosis. One patient was pregnant, and one was breastfeeding. One patient was diagnosed with type two diabetes during hospital care. None of the patients had a known chronic kidney disease before the PUUV infection. Three patients used metformin prior to hospitalization and two patients received insulin during hospital treatment.

A chest radiograph was taken from 75/185 (41%) patients at hospital admission. Radiographs were analyzed by one radiologist for the presence or absence of pulmonary infiltrations or pleural effusion [11]. Chest radiographs were graded as normal, or as having mild, moderate or severe changes as described by Paakkala et al. [11].

Shock was defined as having systolic blood pressures of under 90 mmHg and clinical symptoms of shock such as pale, cold, or clammy skin, rapid breathing, and rapid heartbeat.

All patients provided a written informed consent, and the study with its extensions was approved by the Ethics Committee of the Tampere University Hospital (study codes 97166, 99256, R04180, R15007 and R09206).

### 2.2. Laboratory Determinations

The diagnosis of PUUV infection was made by detecting the typical granular staining pattern of acute infection and/or low avidity of IgG antibodies in immunofluorescence using PUUV -infected Vero E6 cells as antigens, and/or by detecting PUUV IgM antibodies by an “in-house” enzyme-linked immunosorbent assay based on baculovirus-expressed PUUV nucleocapsid protein. The development and use of the above and diagnostic methods have been described elsewhere [12].

Type 2 diabetes is diagnosed based on increased concentration of glycated hemoglobin (HbA1c), fasting plasma glucose, or 2-h postprandial plasma glucose. According to the American Diabetes Association (ADA) and World Health Organization (WHO) criteria, P-Gluc < 7.8 mmol/L is normal in oral glucose tolerance test, P-Gluc 7.8–11.0 mmol/L corresponds to impaired glucose tolerance and P-Gluc > 11.0 mmol/L is diabetic. Random glucose value of >11 mmol/L in a symptomatic patient is diagnostic for diabetes [13]. There are no reference values of what constitutes hyperglycemia during acute illness. For the purpose of the present study, random glucose value <7.8 mmol/L during hospital care was considered normoglycemic, and random glucose level ≥7.8 mmol/L was considered hyperglycemic.

Blood glucose was analyzed from whole blood samples and reported as blood glucose (mmol/L) until August 2003, and as plasma glucose (mmol/L) thereafter. For the analyses, blood glucose values were converted to plasma glucose values by multiplying the blood glucose values by 1.11 [14]. The highest plasma glucose value measured during the hospital treatment was chosen for the analysis, regardless of how many samples per patient were available.

Plasma creatinine was analyzed by Vitros (Johnson & Johnson, Rochester, NY, USA) until the year 1999 and by Cobas Integra (F. Hoffmann-La Roche Ltd., Basel, Switzerland) from thereafter. Blood cell count was determined by automated hematological cell counters (Bayer Diagnostics, Elkhart, IN, USA) and albumin concentrations using routine automated chemistry analyzers. All laboratory determinations were performed by the Laboratory Centre of the Pirkanmaa Hospital District (later named Fimlab laboratories), Tampere, Finland.

Plasma creatinine, C-reactive protein (CRP) and blood count including hematocrit, leukocytes and platelets were measured in 96%–100%, and plasma albumin in 48% of the study subjects. Information on body mass index (BMI) and shock was available from 70% and 90% of the patients, respectively. Minimum or maximum of the values were used in the statistical analysis as indicated in Table 1 and Table 2.

### 2.3. Statistical Analysis

The data are presented as medians and ranges for continuous variables and numbers and percentages for categorical variables. Groups were compared using the Kruskal-Wallis test or Mann–Whitney U test, as appropriate. Bonferroni correction for multiple comparisons was applied in the post-hoc analyses. The Chi-square or Fisher’s exact tests were used to examine differences in proportions. Spearman correlations (r_S_) was used to study the relationship between continuous variables. Multivariate linear regression analyses were performed, taking variables reflecting disease severity (maximum blood hematocrit, leukocytes and CRP, and minimum blood platelet and plasma albumin level, as well as change in weight and duration of hospital stay) as dependent variables, and taking maximum plasma glucose value, BMI, age and sex as independent variables. All analyses were performed using IBM SPSS Statistics version 25 (IBM, Armonk, NY, USA).

## 3. Results

### Clinical, Laboratory and Radiological Findings

Out of 185 patients 134 had maximum plasma glucose <7.8 mmol/L and in 51 patients this was ≥7.8 mmol/L (Table 1 and Table 2). All type 2 diabetic patients were included in the hyperglycemic group (7/51, 14%). Plasma glucose concentration peaked at median day 5 (range 1–25 days) after the onset of first symptom i.e., fever. Two thirds of the patients were men, but the sex distribution did not differ between the groups. Hyperglycemic patients were slightly older and more overweight than normoglycemic patients (Table 1). There was no difference in BMI between the sexes (median BMI of 25.1 for men and 24.9 for women, *p* = 0.917). Upon arrival to the hospital, hyperglycemic patients were more often in shock and they had lower minimum systolic and diastolic blood pressure during hospital care. In addition, they had a greater change in weight and their treatment time at the hospital was longer (Table 1).

Chest radiographs showed pulmonary infiltrations and pleural effusion more often in patients with hyperglycemia compared to those with normoglycemia (Table 1).

As laboratory markers of increased capillary leakage, hyperglycemic patients had higher maximum hematocrit and lower plasma albumin level than normoglycemic patients. Hyperglycemic patients had more severe thrombocytopenia and higher blood leukocyte count, but the maximum CRP value did not differ between the groups (Table 2). 

Hyperglycemic patients had higher maximum plasma creatinine concentration than normoglycemic patients (Table 2). They had glucosuria more often in a dipstick urine test at hospital admission (31% vs. 5%; *p* < 0.001), but there were no differences in the results of dipstick tests for albuminuria and hematuria between the groups (data not shown).

The results remained the same when the seven diabetics in the hyperglycemic group were excluded from the analysis (data not shown).

Plasma glucose level correlated with BMI (r_S_ 0.278, *p* = 0.001), as did many variables reflecting disease severity (Table 3). In multivariate linear regression analysis with maximum plasma glucose concentration, BMI, age, and sex as independent variables, all of the main laboratory variables reflecting disease severity were associated with maximum plasma glucose concentration (Table 4). In contrast, the variables reflecting disease severity were not associated with BMI, age or sex (Supplementary Tables S1–S7).

**Figure 1:**
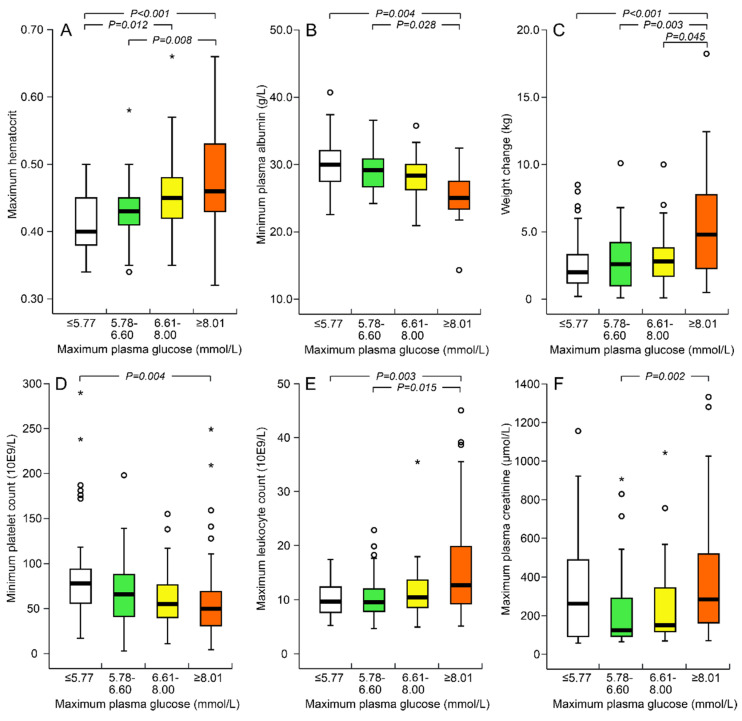
Maximum hematocrit (**A**), minimum plasma albumin (g/L) (**B**), weight change during hospital care (kg) (**C**), minimum platelet count (×10^9^/L) (**D**), maximum blood leukocytes (×10^9^/L) (**E**) and maximum plasma creatinine (µmol/L) (**F**) as grouped according to quartiles of maximum plasma glucose concentration. Boxplots with median, interquartile range, minimum and maximum within 1.5 interquartile range, and outliers displayed as circles and extreme values as asterisks. Bonferroni correction for multiple comparisons was applied in the post-hoc analyses.

When grouping the patients in quartiles of maximum plasma glucose concentration, an apparent dose dependency in relation to the markers of increased capillary leakage, lowest thrombocyte count, and leukocytosis was observed (Figure 1A–E). However, the association of maximum plasma glucose with highest plasma creatinine was more complex: a significant difference was observed between the second and the fourth quartile, while the lowest maximum plasma glucose quartile presented with corresponding creatinine values to the highest fourth quartile (Figure 1F).

## 4. Discussion

In this study, we found for the first time that plasma glucose level was associated with disease severity in patients with acute hantavirus infection. Maximum plasma glucose was related to the clinical, laboratory and radiological markers of increased capillary leakage, and with thrombocytopenia, leukocyte count, and AKI. The present study follows our recent report, in which we found that dipstick glucosuria on admission to hospital predicted a severe course of PUUV infection [10]. Interestingly, our finding on the significance of glucosuria was recently replicated in COVID−19 patients [15].

All patients in the group with plasma glucose <7.8 mmol/L were non-diabetics, while only 14% of patients with plasma glucose ≥7.8 mmol/L were diabetics. We cannot exclude the possibility that some of the patients may have had an underlying impairment in glucose metabolism or undiagnosed diabetes. Nevertheless, a clear difference was detected when patients were grouped according to plasma glucose concentration. In addition, hospitalized patients with PUUV infection often have nausea and vomiting which may have equalized differences in plasma glucose concentration between severely ill and milder cases.

Hyperglycemia in severely ill, non-diabetic patients is believed to be caused by the cytokines (interleukin (IL)-1β and tumor necrosis factor (TNF)-α) that induce insulin resistance, and by hepatic gluconeogenesis driven by glucagon, catecholamines, cortisol, and growth hormone [16]. In PUUV infection, cortisol level is upregulated in the acute phase [17], but according to our unpublished observations, cortisol level does not associate with plasma glucose level in this disease.

Interestingly, it was recently shown in mice that viral infection with murine cytomegalovirus (MCMV), influenza A (INFA) virus and lymphocytic choriomeningitis virus (LCMV) induce production of interferon gamma (IFN-γ) by natural killer (NK) cells, which downregulates insulin receptor transcription in skeletal muscle, causing insulin resistance without glucose elevation [18]. Insulin resistance is compensated by increased pancreatic insulin production, thus preserving glycemic control. The resulting hyperinsulinemia boosts CD8^+^ T-cell mediated antiviral response. However, in obese, pre-diabetic mice compensatory mechanisms are overloaded, and long-term loss of glycemic control ensues. This mechanism links the immune and the endocrine systems [18]. Whether the observed hyperglycemia during acute PUUV infection actually reflects the extent of hyperinsulinemia, which then could be at least partially responsible for the well-documented CD8^+^ T-cell responses during acute PUUV infection [19], remains to be determined.

SARS-CoV-1 is known to induce hyperglycemia by causing dysfunction in pancreatic islet cells [20]. Middle Eastern Respiratory Syndrome (MERS) coronavirus is anchored to host cells via dipeptidyl peptidase-4 (DPP-4) [21] which is responsible for the degradation of incretins such as glucagonlike peptide−1 (GLP-1). In influenza A infection, elevated inflammatory cytokine expression levels are tightly correlated with high levels of blood glucose [22]. Pancreatic enlargement has been detected in SARS-CoV-2 infected patients possibly affecting insulin production and causing hyperglycemia in non-diabetic patients [23]. Thus, multiple mechanisms of virus-induced hyperglycemia exist.

It is currently not known, what causes the glucose elevation in PUUV infection. Similarly to MCMV, INFA and LCMV infections [18,22], cytokine induced insulin resistance and hormonal changes [16] are possible. PUUV infects tissues throughout the body. Although infection induced pancreatitis is usually not detected [24], the possibility of infection induced changes in insulin production cannot be excluded. Like PUUV, LCMV and Ljungan-virus (also called Parechovirus B) use bank vole as a reservoir, and Ljungan virus induces pancreatic islet autoantibody production [25,26]. Sequential or co-infection of PUUV with either one of the two viruses might therefore result in changes in glucose metabolism [26]. Direct molecular mechanisms, as in coronavirus infections, are currently unknown.

In our study, plasma glucose concentration related to the severity of capillary leakage and thrombocytopenia. It is possible, that higher plasma glucose concentration is merely a sign of more severe disease during PUUV infection. However, plasma glucose may also influence the pathophysiological process of PUUV infection by damaging vascular endothelium. Septic bacterial infection and hyperglycemia are known to associate with loss of endothelial glycocalyx, the mechanism of which contributes to vascular dysfunction and coagulation activation [27,28,29]. Sepsis leads to ubiquitous degradation of the glycocalyx, altered endothelial permeability with hypovolemia, hypoalbuminemia, and edema. Several inflammatory mediators are implicated in the pathophysiology and glycocalyx shedding is worsened by hyperglycemia [29]. In mice, short-term hyperglycemia increases permeability of endothelial glycocalyx, an effect that is already evident at 60 min after the beginning of hyperglycemia [28]. Indeed, it has been shown that endothelial glycocalyx degradation fragments are detected during PUUV infection [30], as well as a large number of other markers of endothelial damage, including fibrinogen, von Willebrand factor (vWF), D-dimer, plasma prothrombin fragments (F1 + 2), tissue plasminogen activator (tPA) and IL-6 [31,32,33,34]. An increased platelet consumption on the endothelium is considered to be the mechanism of thrombocytopenia in hantavirus infections [1,35]. Thus, any further damage to the endothelium would worsen thrombocytopenia.

In addition to serving as a link between capillary leakage and coagulation, damage to the glycocalyx may interfere with its role as a regulator of inflammation via the capturing of cytokines. Cytokine storm is a well-known finding in hantavirus infections. Plasma levels of IL-1β, IL-6, IL-1Ra and TNF-α are clearly elevated in acute PUUV infection and urinary IL-6 excretion is associated with the amount of proteinuria of the disease [34,36]. A plausible explanation is that hyperglycemia-contributes to the viral infection induced endothelial damage, resulting in more severe thrombocytopenia, inflammation and capillary leakage.

Diabetes predisposes to AKI. Interestingly, even transient hyperglycemia has been associated with AKI in clinical and experimental settings, the most prominent damage appearing in the renal tubules [8,9,37]. PUUV infects renal tubular cells and acute tubulointerstitial nephritis is detected histologically. It can be speculated that changes in glucose concentration may further aggravate tubulointerstitial injury in PUUV infection [2].

An apparent dose dependency was observed when capillary leakage markers, platelet count, and blood leukocyte count were grouped according to the quartiles of maximum plasma glucose concentration (Figure 1A–E). However, maximum plasma creatinine concentrations were corresponding in the lowest and highest quartiles of maximum plasma glucose concentration (Figure 1F). This discrepancy may be related to impaired gluconeogenesis in the injured proximal tubular cells of the kidney. During fasting conditions, and even under situations of stress, gluconeogenesis in the kidney produces up to 40% of the systemically available glucose [38]. Ischemia reperfusion injury has been found to impair gluconeogenesis in the kidney, a mechanism that was not fully compensated for by the liver [38]. Another possible explanation is that during AKI, anorexia, nausea, and vomiting may have affected the glucose levels. Altogether, the relation between plasma glucose concentration and AKI is complex.

The present study is observational. The measurements of plasma glucose concentrations were based on clinical relevance instead of systematic sampling. A prospective study is needed to confirm the present results. However, our results are supported by previous findings demonstrating that viral infections can affect glucose metabolism [18,20,21,22], and that both infection and plasma glucose concentration may contribute to the pathogenesis of capillary leakage, thrombocytopenia and AKI [8,9,27,28]. Therefore, subtle changes in plasma glucose level in acutely ill patients serve as biomarkers of disease severity in PUUV infection. The present study only included hospital treated patients. Whether the results are applicable to those with a mild disease could not be addressed.

## 5. Conclusions

We have described here a novel finding that plasma glucose levels relate to the clinical, laboratory and radiological markers of increased capillary leakage, thrombocytopenia, inflammation, and AKI in PUUV infection. Further studies are needed to explore both the mechanism of hyperglycemia and the possible pathophysiological role of plasma glucose behind these findings.

## Figures and Tables

**Table 1:** Clinical findings in 185 patients with PUUV infection according to maximum plasma glucose concentration.

	Plasma Glucose < 7.8 mmol/L		Plasma Glucose ≥ 7.8 mmol/L		
	*n* = 134		*n* = 51		
Plasma and Blood Findings	Median/Number	Range/%	Median/Number	Range/%	*p*-Value
Age (years)	40	18–77	48	25–69	0.003
Male/female *	90/44	73/71	33/18	27/29	0.862 ‡
Diabetes	0/134	0	7/51	14	<0.001 †
BMI	24.8	19.8–35.8	27.3	21.0–37.2	0.005
Shock τ	2/119	2	7/46	15	0.002 †
Systolic BP initial (mmHg)	124	90–210	125	60–180	0.594
Diastolic BP initial (mmHg)	80	30–105	78	30–110	0.772
Min systolic BP (mmHg)	116	87–170	110	60–150	0.010
Min diastolic BP (mmHg)	72	46–100	69	36–95	0.044
Weight change (kg) ~	2.6	0.1–10.1	4.8	0.5–18.5	<0.001
Dialysis	4/119	3	5/45	11	0.116 †
Pulmonary infiltrations in chest radiograph (normal/mild/moderate)	37/20/0	65/35/0	7/9/2	39/50/11	0.019 †
Pleural effusion in chest radiograph	15/57	26	10/18	56	0.024 †
Hospital stay (days)	6	1–20	8	3–30	<0.001

* Number or percentage within sex. ‡ Pearson Chi-Square; † Fisher’s exact test, all others Mann-Whitney U test. τ Shock defined as systolic blood pressure <90 mmHg and symptoms of a shock. ~ Difference between highest and lowest weight during hospital care reflecting both capillary leakage and fluid accumulation during oliguric phase. BMI, body mass index; BP, blood pressure.

**Table 2:** Laboratory findings in 185 patients with PUUV infection according to maximum plasma glucose concentration.

	Plasma Glucose < 7.8 mmol/L		Plasma Glucose ≥ 7.8 mmol/L		
Plasma and Blood Findings	Median	Range	Median	Range	*p*-Value
Glucose max (mmol/L)	6.2	4.2–7.7	9.3	7.8–34.1	<0.001
Glucose min (mmol/L)	5.9	4.0–7.7	7.4	4.4–34.1	<0.001
Glucose arrival (mmol/L)	6.2	4.0–7.7	8.7	6.1–34.1	<0.001
Platelets min (×10^9^/L)	66	3–293	50	4–249	0.001
Hematocrit max	0.43	0.34–0.58	0.46	0.32–0.66	<0.001
Albumin min (g/L)	29	21–43	24	11–33	<0.001
Leukocytes max (×10^9^/L)	9.6	4.6–22.8	13.0	5.1–45.0	<0.001
CRP max (mg/L)	74	9–305	79	19–214	0.157
Creatinine max (µmol/L)	151	58–1156	272	71–1499	0.001

CRP, C-reactive protein.

**Table 3:** Correlation coefficients of variables reflecting disease severity with maximum plasma glucose and body mass index (BMI).

	Correlation Coefficient for Maximum Plasma Glucose	*p*-Value	Correlation Coefficient for BMI	*p*-Value
Platelets min (×10^9^/L)	−0.307	<0.001	0.085	0.130
Hematocrit max	0.369	<0.001	0.153	0.006
Albumin min (g/L)	−0.375	<0.001	−0.121	0.150
Leukocytes max (×10^9^/L)	0.262	<0.001	0.094	0.094
CRP max (mg/L)	0.201	0.007	0.166	0.003
Weight change (kg)	0.316	<0.001	0.095	0.096
Hospital stay (days)	0.296	<0.001	−0.017	0.763

Spearman’s correlations. CRP, C-reactive protein.

**Table 4:** Multivariate linear regression analysis for disease severity outcomes, dependent of maximum plasma glucose concentration.

	Maximum Plasma Glucose			
	B	SE	Beta	*p*-Value
Platelets min (×10^9^/L)	−4.083	1.390	−0.251	0.004
Hematocrit max	0.006	0.002	0.270	0.002
Albumin min (g/L)	−0.580	0.181	−0.374	0.002
Leukocytes max (×10^9^/L)	0.931	0.209	0.378	0.000
CRP max (mg/L)	3.073	1.731	0.160	0.078
Weight change (kg)	0.323	0.094	0.308	0.001
Hospital stay (days)	0.322	0.106	0.269	0.003

SE, standard error of the mean; CRP, C-reactive protein.

## Data Availability

Original data is available as supplementary material.

## Supplementary Materials

The following are available online at https://www.mdpi.com/article/10.3390/v13061177/s1. Tables S1–S7: The results of multivariate linear regression analysis with maximum plasma glucose concentration, BMI, age and sex as independent variables and disease severity parameters ad dependent variables.
