# Supplementary material for: The Clinical Presentation of Puumala Hantavirus Induced Hemorrhagic Fever with Renal Syndrome Is Related to Plasma Glucose Concentration
Source: Viruses. 2021 Jun 20;13(6):1177. doi: 10.3390/v13061177 (PMC8235586; doi:10.3390/v13061177)
Supplement: Supplementary file 1 [file viruses-13-01177-s001.zip › viruses-1237155-supplementary.pdf]

viruses MDPI

The abbreviations in the following supplementary tables are: BMI, body mass index; SE, standard error of the mean; CRP, C-reactive protein.

Table S1.

Header	Minimum blood platelet count			
	B	SE	Beta	*p*-value
Maximum plasma glucose	−4.083	1.390	−0.251	0.004
BMI	0.996	0.935	0.93	0.288
Age	0.964	0.288	0.282	0.001
Sex	−8.044	7.414	−0.089	0.280

Table S2.

	Maximum blood hematocrit			
	B	SE	Beta	*p*-value
Maximum plasma glucose	0.006	0.002	0.270	0.002
BMI	0.002	0.001	0.142	0.108
Age	−0.001	0.000	−0.176	0.039
Sex	−0.016	0.010	−0.123	0.135

Table S3.

	Minimum plasma albumin			
	B	SE	Beta	*p*-value
Maximum plasma glucose	−0.580	0.181	−0.374	0.002
BMI	0.213	0.144	0.178	0.144
Age	−0.063	0.045	−0.161	0.167
Sex	−2.302	1.192	−0.218	0.058

Table S4.

	Maximum blood leukocytes			
	B	SE	Beta	*p*-value
Maximum plasma glucose	0.931	0.209	0.378	0.000
BMI	−0.122	0.141	−0.069	0.430
Age	−0.083	0.043	−0.160	0.057
Sex	−0.188	1.115	−0.014	0.666

Table S5.

	Maximum plasma CRP			
	B	SE	Beta	*p*-value
Maximum plasma glucose	3.073	1.731	0.160	0.078
BMI	−0.738	1.199	−0.057	0.539
Age	0.429	0.359	0.106	0.234
Sex	−18.681	9.264	−0.173	0.046

Table S6.

	Weight change			
	**B**	**SE**	**Beta**	***p*-value**
Maximum plasma glucose	0.323	0.094	0.308	0.001
BMI	−0.014	0.064	−0.020	0.832
Age	0.024	0.020	0.104	0.239
Sex	−0.541	0.508	−0.092	0.290

Table S7.

	Length of hospital stay			
	**B**	**SE**	**Beta**	***p*-value**
Maximum plasma glucose	0.322	0.106	0.269	0.003
BMI	−0.075	0.071	−0.096	0.292
Age	0.035	0.022	0.140	0.109
Sex	0.101	0.564	0.015	0.858
